# Ranking United States' Medical Residency Programs: A Systematic Review and Critical Analysis of Doximity’s Residency Navigator Tool

**DOI:** 10.7759/cureus.79579

**Published:** 2025-02-24

**Authors:** Ethan D Paliwoda, Avi A Gajjar, Shrey Patel, Saketh Bhupathiraju, Awinita Barpujari, Raj Swaroop Lavadi, Rida Mitha, Sangami Pugazenthi, James Mooney

**Affiliations:** 1 Albany Medical College, Albany Medical Center, Albany, USA; 2 Department of Neurological Surgery, University of Pittsburgh School of Medicine, Pittsburgh, USA; 3 Department of Surgery, Tufts University School of Medicine, Boston, USA; 4 School of Arts and Sciences, Washington University in St. Louis, St. Louis, USA; 5 Department of Neurosurgery, Drexel University College of Medicine, Philadelphia, USA; 6 Department of Neurological Surgery, Washington University School of Medicine, St. Louis, USA

**Keywords:** doximity, match, medical residency, ranking, reputation, residency

## Abstract

Doximity (Doximity, Inc., San Francisco, CA), a platform for healthcare professionals, aids residency applicants in program ranking decisions, despite debate over its rankings' reliability due to a lack of objectivity. Nevertheless, studies reveal that Doximity’s Residency Navigator (DRN) rankings influence program selection. This study aims to analyze the impact of Doximity rankings on applicant decision-making. A systematic review guided by the Preferred Reporting Items for Systematic Reviews and Meta-Analyses (PRISMA) was conducted and PubMed, Google Scholar, and Web of Science databases were queried to identify DRN-related articles. Covidence (Veritas Health Innovation Ltd, Melbourne, Australia) was used for screening. The review included studies evaluating DRN rankings' usage by residency applicants. A total of eight of 266 screened articles were included. Survey data were analyzed from 5,068 residency applicants: 22% emergency medicine, 16% general surgery, and 12% internal medicine. Common themes encompassed the influence of the program's social media or DRN reputation (7/8), calls for enhanced DRN objectivity (4/8), and the significance of the program's online presence (3/8) and applicants’ mentors (2/8). Perceived inaccuracies included DRN's survey sampling bias and construct validity. Despite applicant perception of inaccuracy, DRN rankings influence program preferences and applicants’ residency program rank lists. This study recommends future research to develop an objective residency ranking system that better aligns with applicant priorities.

## Introduction and background

Matching into a United States (US) medical residency program is highly competitive [1]. Residency programs evaluate numerous factors such as United States Medical Licensing Examination (USMLE) scores, research productivity, grades, awards, and letters of recommendation [2,3]. Medical students also consider program-specific variables, such as research output, resident wellness, mentorship, and case volume when applying to residency [4]. The match is facilitated by the National Resident Matching Program (NRMP). Once students and programs submit rank lists to the NRMP, applicants and programs are matched using a complex algorithm [5]. Given that this is a significant event for trainees, medical students often seek insight from experienced professionals, yet they still rely on other sources of information such as social media networks.

Doximity (Doximity, Inc., San Francisco, CA) is a large networking platform for healthcare professionals, with over 70% of US physicians as members [6]. Doximity's Residency Navigator (DRN) facilitates a medical school graduate’s decision when finalizing their rank list [6]. The DRN includes a resident satisfaction survey, reputation data, and objective data such as alumni publication percentile and top subspecialties that residents match into. Rankings are derived from an alumni-weighted nomination survey of board-certified physicians and are used by healthcare professionals throughout their training [6]. Rankings are used for physician surveys, program reputations, and decision-making by medical students. However, accurate assessment is challenging due to subjective factors and human bias.

The DRN’s methodology has been previously scrutinized due to the lack of scientific criteria in assessing program characteristics such as alumni research productivity and fellowship placements [7]. DRN rankings have been criticized for favoring programs with larger alumni networks, raising concerns about inherent biases that could mislead applicants [7]. Despite this, research has shown that DRN rankings influence applicants' decisions when selecting residency programs [7,8]. This study aims to systematically review the literature on the use and impact of Doximity's annual residency navigator across various medical specialties.

## Review

Methods

Search Strategy

**Figure 1:**
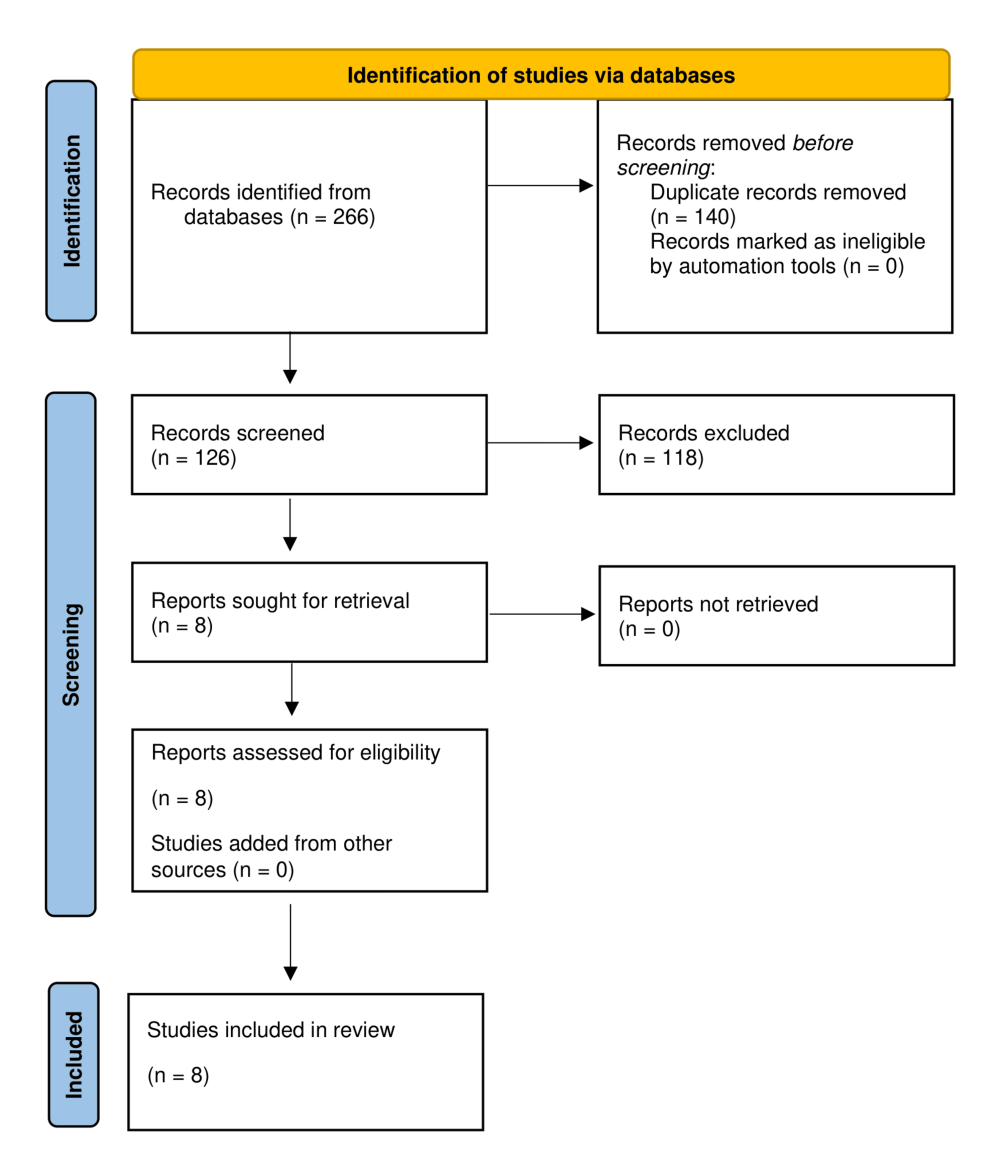
PRISMA 2020 flow diagram. PRISMA: Preferred Reporting Items for Systematic Reviews and Meta-Analyses.

The most recent Preferred Reporting Items for Systematic Reviews and Meta-Analyses (PRISMA) guidelines were followed to conduct a comprehensive literature search (Figure 1) [9]. PubMed, Google Scholar, and Web of Science were queried using relevant search terms, Medical Subject Headings (MeSH), and Boolean search terms for pertinent articles published between 2015 and 2022. The inclusion criteria consisted of studies published in the English language that evaluated the use of DRN rankings by residency program applicants. Conference abstracts, unpublished manuscripts, textbook chapters, non-English manuscripts, and studies straying from the residency applicant population were excluded. Non-English studies were excluded due to feasibility constraints in accurate translation and interpretation within our research framework.

Identified articles were then uploaded to Covidence (Veritas Health Innovation Ltd, Melbourne, Australia) for systematic article storage and management [10]. Reviewers utilized a standardized form to extract pertinent information, including study characteristics (e.g., author, publication year, and journal), participant data, and key findings. Data extraction was cross-verified for accuracy and completeness. Three independent reviewers selected and included all relevant articles. Conflicts were resolved by discussion until a unanimous decision was reached. All duplicate articles were removed. Full-text articles were obtained for those that met the inclusion criteria. Survey data were analyzed using descriptive statistics and thematic synthesis to identify common concerns regarding DRN rankings.

Results

The search process initially revealed 266 articles for further examination. Following the screening, eight relevant studies, comprising experiences from 5,068 residency program applicants, were identified and included in this comprehensive analysis [7,8,11-16]. All the included articles were cross-sectional studies. Among the identified studies, three studies specifically focused on medical residency program applicants, while an equal number concentrated on surgical residency applicants. A detailed breakdown of residency specialties was conducted and presented in Table 1. This analysis encompasses a wide range of medical specialties, with emergency medicine emerging as the most prevalent specialty, constituting approximately 22% of the applicant pool (Table 1). General surgery applicants comprised approximately 16% of the sample and internal medicine comprised approximately 12% (Table 1). Only two studies mentioned sample sizes of osteopathic and non-US medical students in which 134 respondents were identified as coming from osteopathic schools and at least 774 respondents were from international medical schools.

**Table 1:** Medical and surgical specialties included in the review.

Residency specialty	Number of applicants
Emergency medicine	1,114
General surgery	826
Internal medicine	622
Unspecified	397
Orthopedic surgery	372
Anesthesiology	281
Plastic surgery	207
Family medicine	202
Pediatrics	172
Neurology	126
Radiology	108
Psychiatry	100
Pathology	91
Obstetrics & gynecology	75
Dermatology	72
Otolaryngology	65
Physical medicine & rehabilitation	57
Neurosurgery	46
Radiation oncology	41
Ophthalmology	22
Child neurology	19
Medicine/pediatrics	15
Thoracic surgery	15
Vascular surgery	10
Urology	9
Preventative medicine	2
Medical genetics	1
Nuclear medicine	1

Seven articles highlighted that DRN or the social media profiles of the residency programs impacted applicant program preferences. Another three of the included articles highlighted that the official residency program website was the most utilized resource in ranking programs, while two of the articles stated that Doximity was the most utilized resource. Similarly, four articles called for greater objectivity regarding DRN rankings. Despite recognized accuracy and objectivity concerns, four articles mentioned how Doximity was still regarded as an influential resource.

Related to the disruptions to sub-internships and changes in culture following the COVID-19 pandemic, three articles highlighted how applicants began to rely on other sources of information, such as social media, to gauge a program’s reputation and culture. Furthermore, two articles stated that residency programs with a social media presence were considered more accessible, and another three highlighted that residency program social media accounts were informative tools. Meanwhile, another two articles emphasized the value and importance of mentor input in influencing program rank lists. One article found that Doximity was the least important factor that influenced rank lists, but nonetheless still influenced decision-making.

Discussion

Reputation Rankings

The DRN rankings are a well-known resource among residency program applicants, with reputation rankings like those provided by DRN still being considered valuable by many. A survey of 2152 applicants across 24 graduate medical education programs showed that 78% of applicants who reviewed DRN’s reputation rankings found reputation rankings to be valuable, and 79% said that DRN's reputation rankings influenced their application decisions [8]. In addition, another study found that 86% of surveyed applicants used Doximity as their primary social media platform for gathering information about residency programs, even though only 64% had membership access to complete rankings and program data [12].

DRN's popularity is also discussed by other studies. For instance, one study found that DRN was the second most preferred source of residency program reputation by 159 fourth-year medical students applying to emergency medicine, after the advice of mentors and faculty [15]. Nonetheless, there are some exceptions. For example, another study discovered that only 5.5% of general surgery residency applicants used Doximity as their primary source; instead, most preferred the official residency website, Twitter, or Instagram [13]. Similarly, researchers reported that DRN was only the third most popular resource for 47% of integrated plastic surgery residency applicants, trailing the residency program website and Instagram [14]. In addition, another survey found that DRN reputation rankings were the least important factor considered by applicants when evaluated along with other factors such as geographical location and interview experience [7]. An additional survey found that 38% of applicants did not use DRN, of which 77% were unaware of its existence [8].

One study found that 73% of orthopedic surgery residency applicants used DRN, but its mean quality ranking was only 3.5 on a five-point Likert scale [16]. Rather, many applicants preferred an open-edit Google document with program information, leading almost half of them to apply to fewer programs [16]. Further, another study reported that on a 0-100 scale, respondents’ perceived mean accuracy score of DRN rankings was 41 [7]. Thus, while DRN is a popular resource, its lack of comprehensive and reliable program information may not always meet applicants' needs. As such, anonymous information exchanges on Doximity, like those on Reddit, should be considered with caution in addition to other sources of program information [15]. Despite shortcomings in perceived accuracy, Doximity rankings do seem to influence applicant rank list decisions; one study found that 26% of the students who utilized DRN rankings added programs to their list while 9.8% removed programs [7].

Due to the COVID-19 pandemic, social media has become increasingly important for connecting with residency programs [14]. Specifically, many plastic surgery residency applicants were unable to complete multiple sub-internship rotations due to COVID-related limitations [14]. As a result, Doximity and other social media platforms served as a proxy for evaluating residency programs, despite lacking important evaluation aspects such as allowing applicants to assess a program’s culture, alignment with their goals, and objective measures of prestige. For applicants of specialties most disrupted by the COVID-19 pandemic, the utility of other information avenues, such as Doximity, became increasingly important [15].

Doximity Pitfalls

Program directors have long expressed concerns about the use of reputation data to rank residency programs [17]. Prior research and clinicians have reinforced these worries, arguing that Doximity’s reputation scores are undermined by their reliance on data that are not objectively measured or tied to tangible outcomes [11,17,18]. These rankings do not correlate with additional objective data such as board pass rate and publications authored by program alumni [18].

One study detailed the perceived inaccuracies of the DRN, including both the construct and measurement validity of the survey utilized by Doximity as well as the sampling bias toward physicians who do use Doximity [11]. Concerns arise that these physicians are restricted to commenting on those programs where they have experience [11]. Subsequently, a push for an increase in the use of Doximity by a particular program’s staff may positively, yet subjectively, influence a program’s ratings [11].

One limitation of Doximity's methodology for determining program rank is that it heavily relies on a peer nomination system. Additionally, the rankings for residency programs show significant variation annually attributable to Doximity’s survey methodology and lack a composite trend across years [19].

Previous investigations have shown that larger residency programs tend to receive higher rankings on Doximity, but this observation may be influenced by greater funding availability [20]. Applicants should not be misled by program size or funding, as these do not necessarily correspond to the quality of training and mentorship provided by a program. Therefore, residency applicants should not solely rely on Doximity's rankings to assess the quality of a program.

In 2014, leaders of major emergency medicine organizations released a consensus statement expressing their opposition to the Doximity rankings [21]. They also drew attention to critical issues with Doximity’s polling techniques, noting a potential sampling bias since the surveys were completed by members recruited via social media [21]. It was also noted that it is unlikely that physicians have detailed knowledge of programs and facilities outside of their home institutions [21].

Additionally, they warned about the potential public health implications of relying on reputation rankings as people with medical emergencies may bypass the nearest emergency department for one with a higher ranking [21]. These concerns are relevant not only to emergency medicine but also to all medical specialties. Therefore, residency applicants must approach program rankings with caution and consider multiple factors when making their decisions. Although program reputation is just one aspect of the decision-making process, applicants may potentially believe that this program characteristic will affect their future career trajectories [22]. Additionally, some organizations such as the Alliance for Academic Internal Medicine (AAIM) discourage the use of Doximity due to their flawed methodology and unscientific approach [23].

Suggestions for the Future

Accurate and objective data on residency training programs can benefit all parties involved, including students, residents, attendings, and program directors. Residency applicants should consider objective program characteristics that align with their training goals, such as case volume, operating room time, board exam pass rates, setting, research output, fellowship match rates, alumni career outcomes, and resident satisfaction surveys designed with validated constructs. By doing so, applicants can select a program that best fits their unique needs.

Doximity currently calculates reputation rankings using data from the preceding three years, but the reputations of residency programs should be a lifelong characteristic, not subject to vast yearly variations. A better approach may encompass using a weighted average across multiple years instead of relying on new survey data each year, which can help reduce the large year-to-year variation frequently observed.

This study is not without its limitations. Using specific search criteria may have inadvertently excluded relevant articles from the preliminary search, before screening. Of the included articles, emergency medicine residency program applicants were overrepresented compared to the actual sample distribution seen across all residency programs in the United States. Similar literature does not yet exist in sufficient quantity encompassing other specialties, preventing analysis of utilization differences between applicants to different specialties. The specialty representation here was contingent on the response rate of the surveys created by each study’s authors and their specialty of interest, thereby introducing a potential element of bias. Most included articles did not specify the school type of the applicants. Thus, these findings are more applicable to allopathic medical school graduates; meanwhile, osteopathic and international medical graduates were not adequately represented and discrepancies in utilization between these graduate types could not be assessed. Moreover, many published studies cited in the text are dated prior to the COVID-19 pandemic. We recognize that applicant and program behavior along with Doximity use may have shifted. Finally, a majority of the articles discussed Doximity in a mixture of other online resources for residency rankings; therefore, the results may be more representative of the holistic influence of social media on residency rankings, as opposed to the specific influence of Doximity.

Graduate medical education programs and prospective residency applicants, working together with Doximity Inc., should aim to agree on quantifiable training outcomes and make these educational metrics freely available [7]. Alternatively, internal ranking systems within each specialty’s community could be established. This would help ensure that residency applicants can make informed decisions about their training programs and improve the overall quality of graduate medical education.

## Conclusions

The authors of this study have highlighted the impact of DRN rankings on applicant program rank lists. Despite the high reliance on DRN rankings, a more objective approach to determining program quality is needed due to applicants’ reservations about the DRN’s accuracy. While Doximity may not be as widely used as other social media platforms, it remains a highly regarded indicator of a program's reputation among applicants who utilize it. To better serve applicants, an objective, standardized ranking system incorporating qualitative and quantitative metrics should be developed.
